# Flexural Performance of an Innovative Girder-to-Pier Joint for Composite Bridges with Integral Piers: Full-Scale Test

**DOI:** 10.3390/ma18051157

**Published:** 2025-03-05

**Authors:** Wei Xie, Binju Zhang, Litao Yu, Qingtian Su, Fawas O. Matanmi

**Affiliations:** 1 Department of Bridge Engineering, Tongji University, 1239 Siping Road, Shanghai 200092, China; 2011152@tongji.edu.cn (W.X.); 2393186@tongji.edu.cn (F.O.M.); 2 Tongxiang Transportation Construction Investment Group Co., Ltd., Jiaxing 314500, China; 15867432259@163.com (B.Z.); 18943830932@163.com (L.Y.); 3 Shanghai Engineering Research Center of High Performance Composite Bridges, Shanghai 200092, China

**Keywords:** bridge engineering, composite bridges, hogging moment zone, girder-to-pier joint, full-scale test, UHPC, crack initiation and propagation, elastic flexural capacity

## Abstract

To reduce the maintenance requirements during the service life of highway bridges and enhance the cracking resistance of concrete slabs in the hogging moment zone of continuous composite girders, this paper proposes an innovative girder-to-pier joint for composite bridges with integral piers. Compared to the existing ones, this new joint has structural differences. The middle part of the embedded web is hollowed out to facilitate the construction, and the upper and bottom flanges of the steel girder within this joint are widened. Moreover, cast-in-place ultra-high-performance concrete (UHPC) is applied instead of normal concrete (NC) only on the top surface of the pier. A full-scale test was carried out for this new joint to evaluate the load–displacement relationship, load–strain relationship, crack initiation, and crack propagation. Compared with the numerical simulation results of the reference engineering, the test results demonstrated that the proposed joint exhibited excellent flexural performance and cracking resistance. This paper also proposes a calculation method for the elastic flexural capacity of the girder-to-pier joint incorporating the tensile strength of UHPC, and the calculated result was in good agreement with the experimental result.

## 1. Introduction

Bridge bearings are susceptible to issues such as deflection, separation, excessive shear deformation, and cracking [1] during their service life, which can cause the bridges to deviate from their original design conditions, thereby affecting their safety and durability. Bearing maintenance constitutes a significant portion of the maintenance of bridges. For small- and medium-span bridges, the large number of bearings leads to high costs. Eliminating the bearings or reducing the number of bearings can minimize the number of vulnerable components, thereby improving the durability of the bridges and providing a more economical solution. For spans ranging from 25 m to 40 m, the application of prefabricated steel–concrete composite girders offers several advantages as follows: (i) reducing the weight of concrete slabs potentially affects the support settlements; (ii) eliminating the necessity for prestressing or post-tensioning of the concrete slab; and (iii) shortening the construction time [2]. Moreover, compared with concrete girders and steel girders, steel–concrete composite bridges benefit from steel’s tensile strength and concrete’s compressive strength in the sagging moment regions of continuous girders [3]. Therefore, this paper proposes a solution that involves the prefabrication of steel–concrete composite girders in a factory, followed by the integration of the girders and piers on site. As a rigid frame system, structural integrity can be enhanced, thereby improving earthquake resistance.

### 1.1. Girder-to-Pier/Abutment Joint

**Figure 1:**
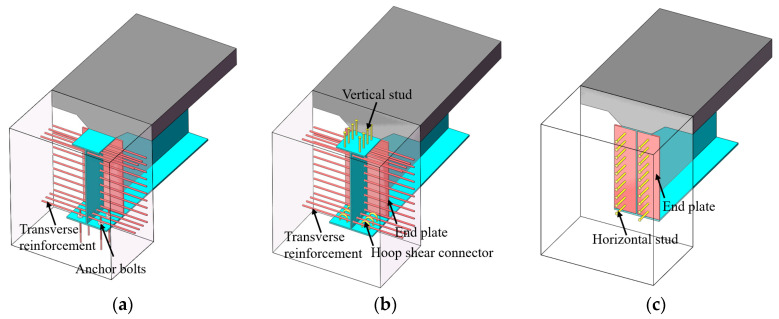
Several types of joints were recommended in the design guidelines of integral steel–concrete composite bridges: (**a**) type I; (**b**) type II; (**c**) type III.

Due to the elimination of bridge bearings, the loads on the superstructure are directly transferred to the piers, making the mechanical performance of the girder-to-pier joint particularly critical. This area is also one of the primary areas of research on similar bridges, both domestically and internationally. Several types of joints were recommended in the design guidelines for integral steel–concrete composite bridges. In the United States [4,5,6], anchored bolts or temporary supports were employed to erect steel girders, with the web holes designed to accommodate the reinforcement in the concrete abutment, as illustrated in Figure 1a. The Steel Construction Institute (SCI) [7] recommended joints in which vertical studs are welded on the top flange, hoop shear connectors are welded on the bottom flange, and the transverse reinforcement is accommodated through the web holes, as illustrated in Figure 1b. The European Commission [8] established the INTAB project to conduct experimental research and theoretical analysis on their mechanical performance, and the joint specified in the design guidelines is illustrated in Figure 1c, where horizontal studs welded on the end plates were used to facilitate the connection between the main girder and concrete cross girder, as well as to transfer the shear forces.

Building on this, several researchers proposed additional joint designs in detail and examined their mechanical performance. Kim et al. [9] carried out an experimental study on six girder-to-abutment joints, and the test results showed that the application of stud shear connectors and perfobond rib shear connectors can enhance the stiffness, crack resistance, and flexural performance of the joint. Lampe et al. [10] experimentally validated the load-bearing capacity of three girder-to-abutment joints and provided several structural recommendations. Briseghella [11], Abbiati [12], and Liang [13] proposed new girder-to-pier or abutment joints and investigated their failure modes and ultimate bearing capacity through an experimental study. Fan et al. [14] studied the mechanical behavior of the girder-to-abutment joint through numerical simulation and conducted a parametric study on the effects of the strength of concrete and steel, as well as the thickness of steel plates. The existing study [9] indicated that the joint exhibited a relatively low initial cracking load, with cracks propagating rapidly once initiated. In practical applications, these cracks could cause corrosion damage and, consequently, reduce the durability of the bridge due to the run-off salt water leaking [15,16]. Additionally, constructing reinforcement within the joint can be challenging.

### 1.2. Measures to Mitigate the Cracking of Concrete Slabs in the Hogging Moment Zone

In response to the issues that concrete slabs in the hogging moment zone of continuous composite girders tend to crack, scholars both domestically and internationally have conducted extensive studies and proposed relevant countermeasures. One approach involves adjusting the concrete pouring sequence in the composite girders to mitigate the tensile stresses of the concrete slabs in the hogging moment zone [17], or adopting a simple-for-dead-load-and-continuous-for-live-load (SDCL) system to mitigate the hogging moment [18,19]. However, these methods cannot induce compressive stresses in concrete slabs in the hogging moment zone. Another approach is to introduce initial prestresses in the concrete slabs, for example, to prestress tendons [20]. However, implementing prestressing systems in the hogging moment zone presents significant construction challenges. Another example is to lift and lower the continuous composite girders to directly generate compressive stresses in the post-cast concrete slabs, but this technique is cumbersome and may lead to uneven stress distribution in the section [21]. A further method is to incorporate crack-resistant materials in the concrete slabs in the hogging moment zone to limit crack initiation and propagation [22,23,24,25], such as engineered cementitious composites (ECCs), steel fiber-reinforced concrete (SFRCs), UHPC, etc. Among these materials, UHPC has superior tensile properties, penetration resistance, and durability [23,26] and has emerged as a highly recommended material in civil engineering in recent years.

**Figure 2:**
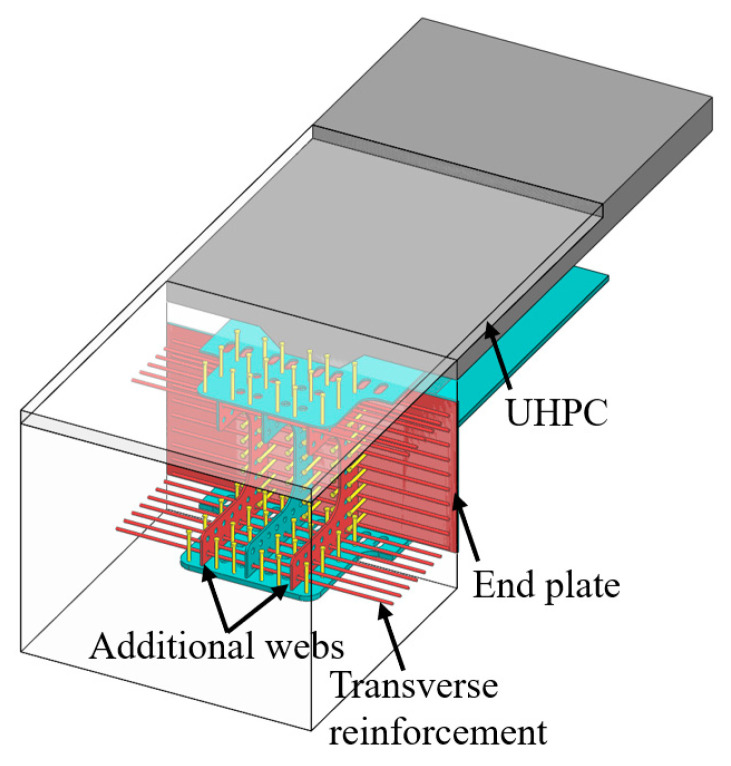
Proposed joint.

Taking one bridge in the Zhejiang Province as the case study, this paper proposes an innovative girder-to-pier joint for composite bridges with integral piers. Compared with existing girder-to-pier or abutment joints, this new joint has structural differences. As illustrated in Figure 2, the middle part of the web is hollowed out to facilitate the construction of reinforcement within the joint. Additional webs are added on both sides of the extended web of the main girder in the transverse direction, and web holes are set to accommodate transverse reinforcement. The upper and bottom flanges of the steel girder within this joint are widened. To enhance the crack-resisting capacity of the joint, UHPC is incorporated for the concrete slabs. For economic efficiency, cast-in-place UHPC is applied instead of NC only on the top surface of the pier.

The paper begins with the introduction of the reference engineering and the finite element analysis to study its overall mechanical performance, proceeds to conduct a full-scale test for the proposed joint, and the test results are subsequently analyzed and evaluated. Finally, a calculation method for the elastic flexural capacity of the girder-to-pier joint, incorporating the tensile strength of UHPC, is proposed.

## 2. Reference Engineering

### 2.1. Bridge Layout

**Figure 3:**
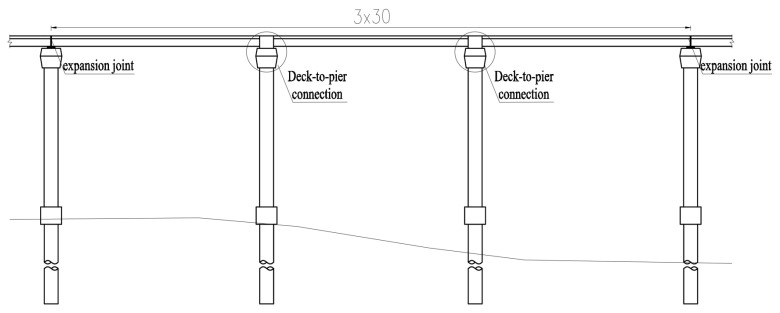
Hybrid girder bridge system and hybrid girder component.

**Figure 4:**
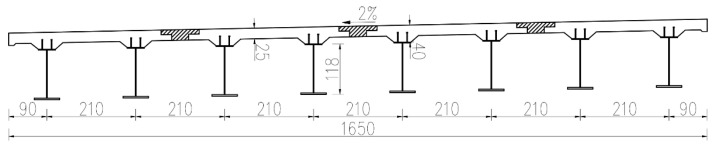
Standard cross-section (unit: cm).

As illustrated in Figure 3, the span layout of the reference engineering is 3 × 30 m, with the main girders and the middle piers integrated. The single-width bridge is 16.5 m wide and consists of four π-shaped composite girders, as depicted in Figure 4. The concrete slabs are C50, with a thickness of 25 cm, thickened to 40 cm at the I-shaped steel girders. The UHPC layer with a thickness of 10 mm and a length of 6 m is applied on the top surface of the middle piers. The height of the steel girder is approximately 1.18 m, with a spacing of 2.1 m between the two steel I-girders. The width (or height) of the top flange, the bottom flange, and the web is 400 mm, 600 mm, and 1124 mm, respectively. The thicknesses of the top flange and the web are both 16 mm, while the thickness of the bottom flanges is 22 mm in the mid-span region and 38 mm near the middle supports.

The construction process of the reference project is as follows: steel girders are processed in the steel processing plant, transported to the prefabrication site, and then concrete slabs are poured to form the π-shaped composite girders; subsequently, the entire structure is moved to the bridge site, and the composite girders are installed. Once the main girders are erected in position, the longitudinal joints between the π-shaped composite girders are poured first, followed by the concrete cross girders at the middle piers, and, finally, the UHPC is poured on the top surface of the middle piers.

**Figure 5:**
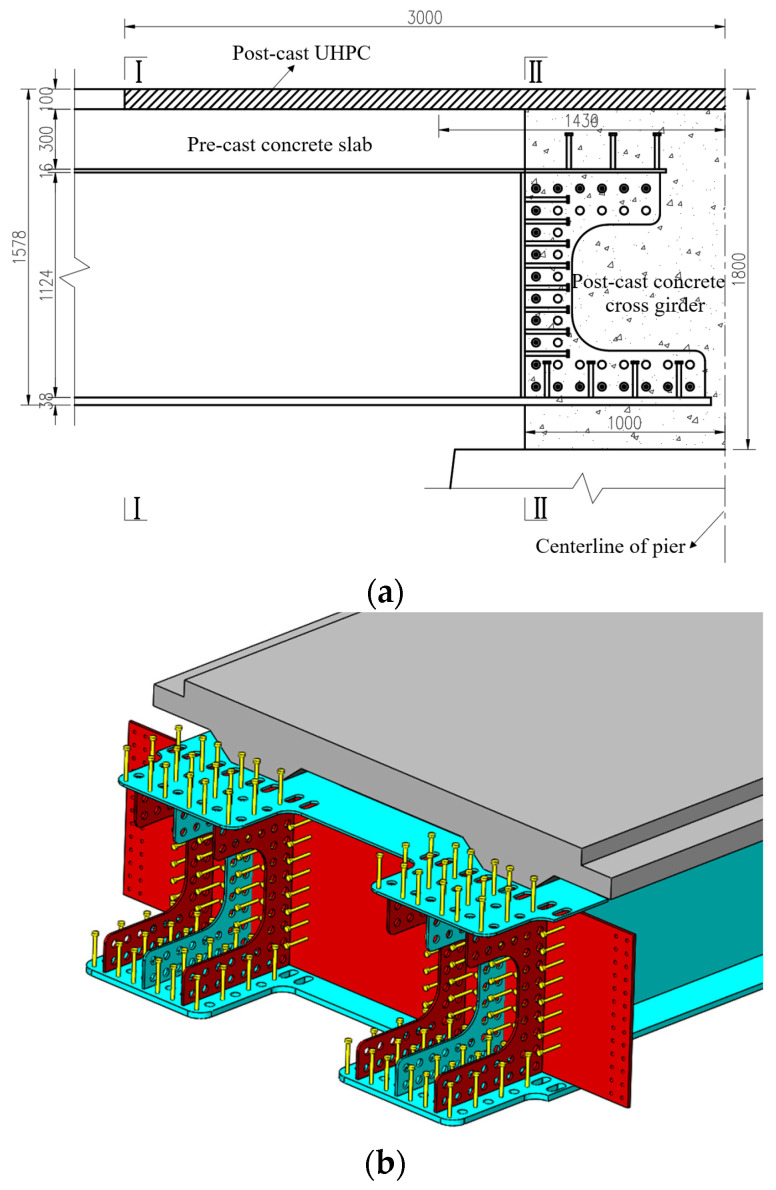
Girder-to-pier joint (unit: mm). (**a**) Elevation of the joint. (**b**) Arrangement of steel components of the joint.

The girder-to-pier joint is illustrated in Figure 5. A 20 mm thick vertical end plate is placed at the interface between the concrete cross girder and the I-shaped steel girder, with stud connectors to provide a reliable connection. The top and bottom flanges of the steel girder extend 70 cm and 90 cm, respectively, to be embedded in the concrete cross girder, and the width of the top and bottom flanges is increased to 100 cm. Holes with a diameter of 60 mm are incorporated on the top and bottom flanges to facilitate the concrete construction. The web extends into the concrete cross girder, with its middle part hollowed out. Three sides of the web are connected to the extended top and bottom flanges and the end plates. Two rows of holes with a diameter of 50 mm are incorporated on the web. Additional webs with the same dimensions are added to each side of the extended web, with a row of transverse reinforcement passing through the holes to enhance the connection between the concrete and the I-shaped steel girder.

### 2.2. Finite Element Analysis

A finite element model was developed by Ansys 2020 to analyze the overall mechanical properties of the reference engineering bridge for the specimen design. The finite element calculation mainly considered the first-phase dead load, the second-phase dead load, the traffic load, and the overall temperature effects. Load combinations were carried out according to the serviceability and ultimate limit states based on the JTGD60-2015 [27]. Figure 5a, sections I and II are the interface between UHPC and NC and the interface between the concrete cross girder and the main girder, respectively. They both exhibit sudden changes in stiffness, making them the weak sections of the structure that require attention. The calculation results, based on the elastic material assumptions, are listed in Table 1. The tensile stresses of the concrete on the top surface at section I under the serviceability and ultimate limit state are 3.8 MPa and 4.6 MPa, respectively, while those of section II are 7.5 MPa and 9.3 MPa, respectively. The calculation results indicate that the tensile stress of concrete is relatively high. The load-resisting capacity and crack-resisting of the girder-to-pier joint with UHPC in the hogging moment zone need to be further verified through testing to ensure compliance with the design requirements.

## 3. Experimental Program

### 3.1. Specimen Design and Manufacture Process

**Figure 6:**
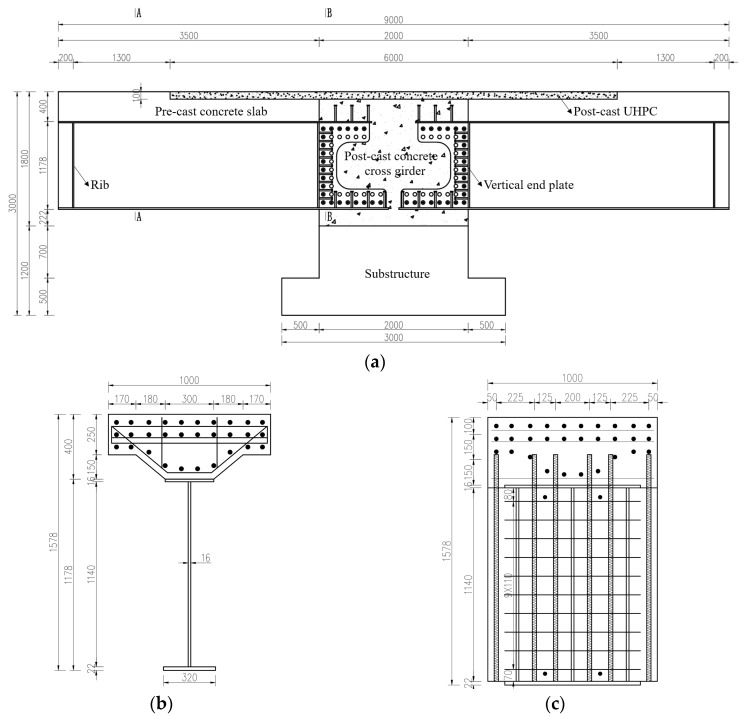
Layout of the specimen (unit: mm). (**a**) Elevation of the specimen. (**b**) Section A-A. (**c**) Section B-B.

**Figure 7:**
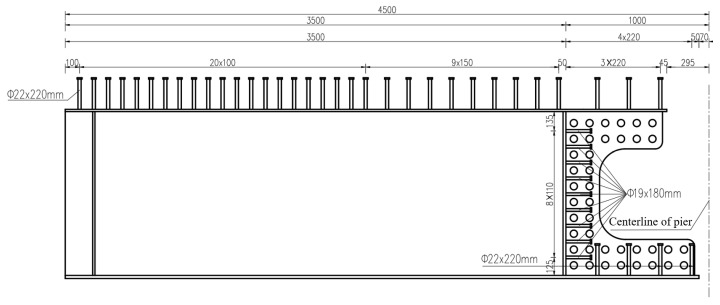
Arrangement of stud connectors (unit: mm).

A full-scale model of a segment of the hogging moment zone was used for the static tests to directly access the girder-to-pier joint. The object of the study was an I-shaped composite girder with a length of 9 m located in the hogging moment zone of the real bridge. The longitudinal cantilever length of the specimen was determined based on the ratio of the flexural moment to the shear force at the middle support under the dead load and the traffic load. Due to the limitation of the lifting capacity of the laboratory, the dimensions of the cross-section were adjusted according to the principle of equivalent flexural stiffness, and the final section is shown in Figure 6. The concrete slab was C50 with a thickness of 25 cm, thickened to 40 cm at the steel I-girder. The UHPC layer with a thickness of 100 mm and a length of 6 m was cast on the top surface of the middle pier. Three layers of longitudinal reinforcement were accommodated in the concrete slab. The diameters of the longitudinal reinforcement in the top layer and the remaining longitudinal reinforcement were 20 mm and 25 mm, respectively. The arrangement of longitudinal reinforcement of the specimen was the same as that of the real bridge. The diameters of the transverse reinforcement in the prefabricated concrete slab and in the cast in situ UHPC layer were 16 mm and 20 mm, respectively. The diameter of the transverse perforated reinforcement in the girder-to-pier joint was 20 mm, and that of the vertical reinforcement was 25 mm. The size of the stud connectors on the upper and bottom flanges was ϕ22 × 220 mm, while that on the vertical end plate was ϕ19 × 180 mm. The arrangement of the stud connectors is shown in Figure 7.

The specimen manufacturing process was similar to the actual bridge construction process, that is, (1) after being welded and assembled in the steel processing plant, the steel girders were moved to the fabrication yard; (2) the concrete slabs and substructure were poured separately and subsequently assembled once the concrete reached early strength, followed by the pouring of the concrete cross girder; (3) the reinforcement in the top layer was tied, followed by the pouring of the top-surface UHPC, and the specimen was cured for 28 days after the construction was completed.

### 3.2. Experimental Setup

**Figure 8:**
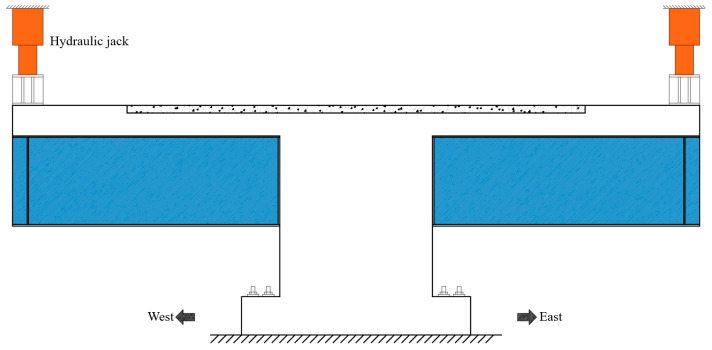
Loading setup.

**Figure 9:**
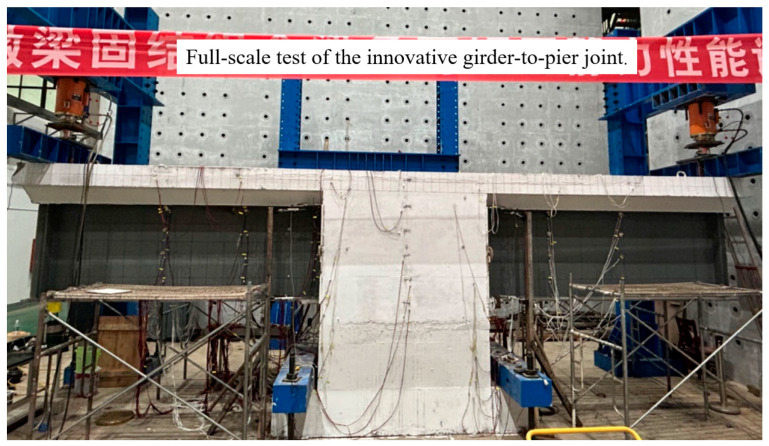
Loading condition on site.

The loading setup is illustrated in Figure 8. A high-strength mortar was placed between the specimen and the ground to ensure horizontal alignment and uniform force distribution. The load was applied to the specimen from the hydraulic jacks via a distribution girder. Two hydraulic jacks were used for synchronous monotonic loading on both sides of the specimen. Preloading was performed prior to formal loading to verify that the loading setup and measurement equipment were working correctly and to compact any gaps between the components. Figure 9 depicts the loading conditions on the site.

**Figure 10:**
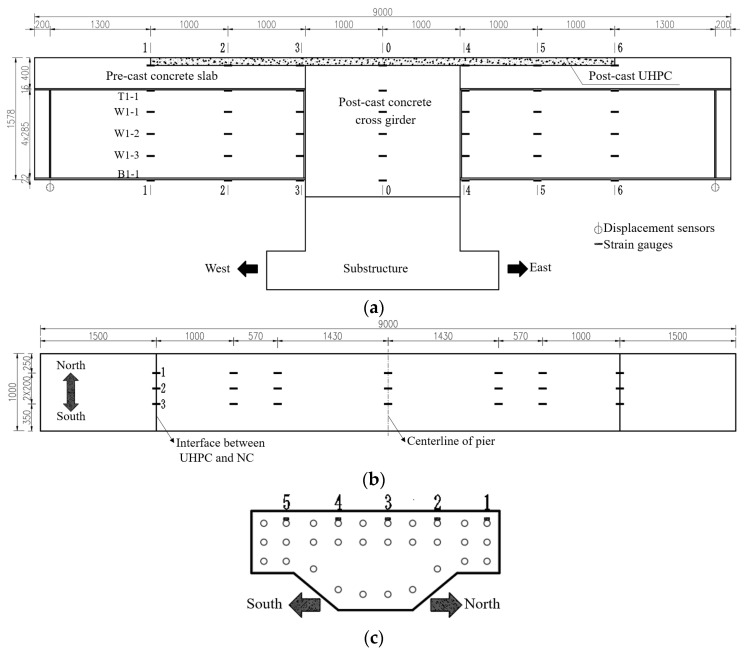
Layout of measurement points: (**a**) measurement points on the side of the specimen; (**b**) measurement points on the top surface of the specimen; (**c**) measurement points on the longitudinal reinforcement.

The layout of the measurement points is illustrated in Figure 10, with seven representative sections (0~6) selected for strain measurements. The strain was monitored on the longitudinal reinforcement, the steel plates, and the concrete slabs. The displacement was measured at both loading points. Following specimen cracking, a crack width gauge with an accuracy of 0.01 mm was employed to measure the width of cracks, and the initiation and propagation of the cracks were manually recorded.

### 3.3. Material Properties

Table 2 lists the material properties of the reinforcement and steel plates, which were determined through tensile tests. For C50 concrete, specimens with dimensions of 150 × 150 × 150 mm and 150 × 150 × 300 mm were, respectively, used for testing the compressive strength and elastic modulus. Similarly, specimens with dimensions of 100 × 100 × 100 mm and 100 × 100 × 300 mm were, respectively, used for testing the compressive strength and elastic modulus of UHPC. Dog-bone-shaped specimens were used for testing the tensile strength of UHPC. The material properties of concrete are listed in Table 3. Table 4 lists the material compositions of 1 m^3^ UHPC, which contains 2.5% steel fiber by volume. The steel fiber has a length of 13 mm and a diameter of 0.2 mm.

## 4. Experimental Results

### 4.1. Failure Process

The specimen was intact and in an elastic state at the initial stage of loading. At a load of 500 kN on each side, first cracks initiated on the side of the NC layer at the cantilever sections (section 3 and section 4), with an initial width of 0.03 mm. The load corresponding to the initiation of the cracks on the top surface of the UHPC layer was 540 kN. As the load increased from 600 kN to 1050 kN, the crack width expanded from 0.05 mm to 0.2 mm. At this moment, the reinforcement and steel beams of the specimen were still in an elastic state. At a load of approximately 2059 kN, the longitudinal reinforcement in the top layer at section 3 and section 4 began to yield, leading to the termination of the test. At this point, the width of the cracks on the top surface of the UHPC layer exceeded 0.4 mm, surpassing the serviceability limit state threshold. The maximum vertical displacement of the loading point was approximately 18 mm. Throughout the entire loading process, there was no obvious slippage or separation between the concrete slab and the I-shaped steel girder.

### 4.2. Load–Displacement Curve

**Figure 11:**
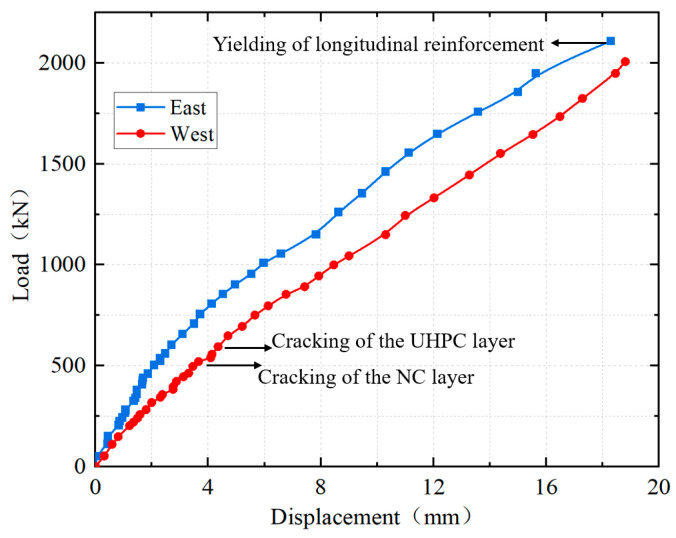
Load–displacement curves.

The load–displacement curves of the loading points are shown in Figure 11. It can be seen that the load–displacement curves at the two ends did not completely coincide, primarily due to the dimensional deviations in the specimen manufacturing and errors in the loading test. The load–displacement curves indicated that the stiffness of the specimen remained relatively high from the beginning to the end of the test. The load–displacement curve of the specimen remained linear up to 500 kN and showed nonlinear characteristics after the load exceeded 500 kN. This was mainly because cracks initiated on the side of the NC layer beneath the UHPC layer at a load of 500 kN. Subsequently, the cracks sequentially initiated and propagated in multiple locations, including the top surface of the UHPC layer, the interface of UHPC-NC, and the central plane of the girder-to-pier joint. As a result, the slope of the load–displacement curve gradually decreased, demonstrating that the specimen’s stiffness progressively diminished as the crack propagated.

### 4.3. Strains of Longitudinal Reinforcement

**Figure 12:**
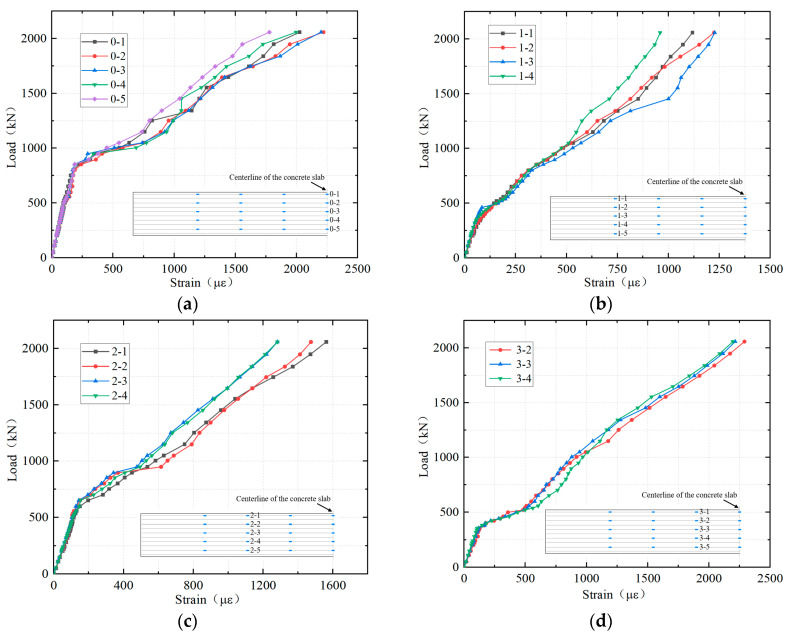
Load–strain curves of the longitudinal reinforcement: (**a**) Section 0; (**b**) Section 1; (**c**) Section 2; and (**d**) Section 3.

The load–strain curves of the longitudinal reinforcement in the top layer in the hogging moment zone are shown in Figure 12. Due to the symmetry of the structure, only the test results of sections (0–3) are presented in this paper. The layout of the measurement points of the reinforcement was shown in Figure 10c. The relationship between the load and the strain of the longitudinal reinforcement of each cross-section can be divided into three phases. During the initial loading stage, the strain of reinforcement was linearly proportional to the load, indicating that the entire structure was in the elastic state; then, there was a sudden increase in the reinforcement strain, primarily due to the cracking of the concrete slab. Consequently, the concrete lost its tensile capacity, transferring the tensile stresses that were initially shared by both the concrete and the reinforcement entirely to the reinforcement. Furthermore, the occurrence of concrete cracking resulted in a downward shift of the neutral axis of the section. With the increasing load, the reinforcement strain continued to rise, but compared to the initial loading stage, the slope of the load–strain curve during this stage was obviously smaller, primarily due to the reduction in the stiffness of the section caused by concrete cracking. When the load reached approximately 2058.7 kN, the longitudinal reinforcement strain at the top of Section 3 (the cantilever root section) exceeded 2233 με, indicating that the reinforcement had just yielded in tension. In addition, the strain distribution of the reinforcement across each section was uniform, and there was no shear lag effect, demonstrating that the full width of the section was effective.

**Figure 13:**
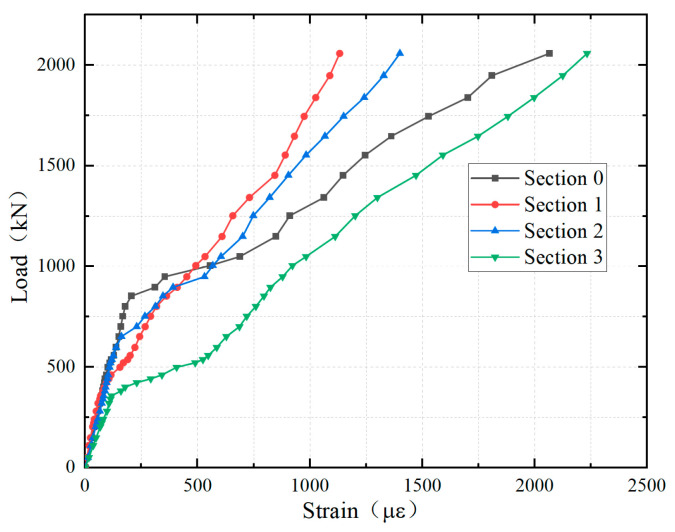
Comparison of reinforcement strains in different sections.

Figure 13 presents the average values of longitudinal reinforcement strains for each section. As previously analyzed, the sudden increase in the reinforcement strain can be attributed to the concrete cracking, with the released tensile stress being transferred to the reinforcement. Thus, the order of cracking in different sections can be inferred from where the strain of longitudinal reinforcement suddenly increased. The earliest cracking occurred at Section 3 (the cantilever root section), primarily because it experienced the largest flexural moment, and there was a sudden change in structural stiffness at this location. Subsequently, cracking occurred at Section 1 (the UHPC-NC interface), mainly because the joint interface was significantly weaker in tensile properties compared to the continuously cast section due to substrate discontinuities and the lack of special processing at the interface. As the load increased, Section 2 and Section 0 (the central plane of the girder-to-pier joint) cracked in sequence.

### 4.4. Strains of Steel Plates

**Figure 14:**
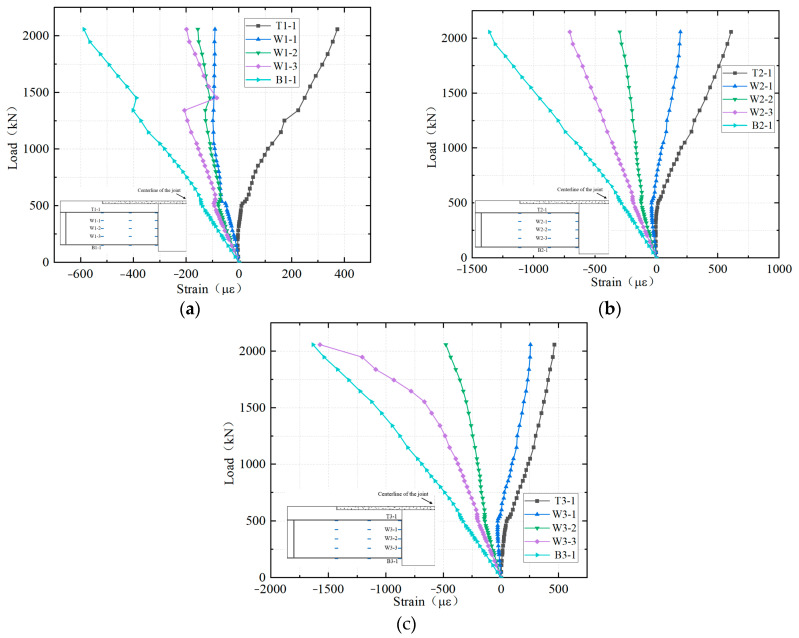
Load–strain curves of the steel plates: (**a**) Section 0; (**b**) Section 1; (**c**) Section 2.

Figure 14 presents the strains on the steel plates. The layout of the measurement points on the steel plates is shown in Figure 10a. Overall, the top flange measurement point T1 and the web measurement point W1 were subjected to tension, while the web measurement points W2-3 and the bottom flange measurement point B1 were subjected to compression, indicating that the neutral axis of each section was located within the web of the steel girder. During the initial stage of loading, the linear correlation between the load and the strain of steel plates indicated that the entire structure was in the elastic state. At a load of 500 kN, the load–strain curves at each section exhibited slight deviations, primarily due to the appearance of the first crack in the NC layer beneath the UHPC layer. It was observed that the strain at the measurement points under compression abruptly decreased, while the strain at the measurement points under tension abruptly increased, owing to the downward shift of the neutral axis of the section caused by concrete cracking. With the increasing load, the slope of the load–strain curve of the web measurement point W3-3 continued to decrease, primarily due to the widening of existing cracks, the formation of new cracks in previously uncracked areas, and the decrease in crack spacing, which collectively led to a gradual decrease in the structural stiffness. When the load reached 2059 kN, the compressive strain of the bottom flanges of the cantilever root section (section 3) reached 1635 με, which indicated that it had not yielded.

### 4.5. Cross-Section Strain Distribution

**Figure 15:**
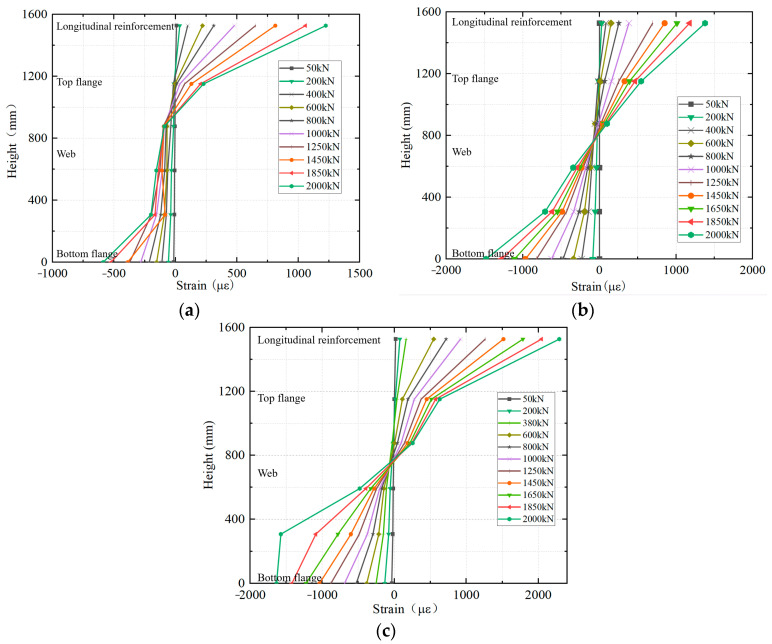
Cross-section strain distribution: (**a**) Section 0; (**b**) Section 1; (**c**) Section 2.

The strain distribution shown in Figure 15 used the bottom surface of the bottom flanges as a zero reference point. Through an analysis of the strain distribution along the height direction of each section of the composite girder under various loading levels, it can be observed that during the initial stage of loading, the strain distribution of each section was linearly proportional to the height of the section, aligning well with the plane section assumption of elastic girder theory. There was a sudden increase in the strain of the longitudinal reinforcement, primarily because the tensile stress was shared by both the reinforcement and concrete before cracks appeared. However, once the concrete cracked under the hogging moment, the tensile stress in this area was carried solely by the reinforcement. As the load continued to rise, the concrete slab ceased to contribute structurally, with a gradual downward shift of the neutral axis of the section.

### 4.6. Cracking Patterns

**Figure 16:**
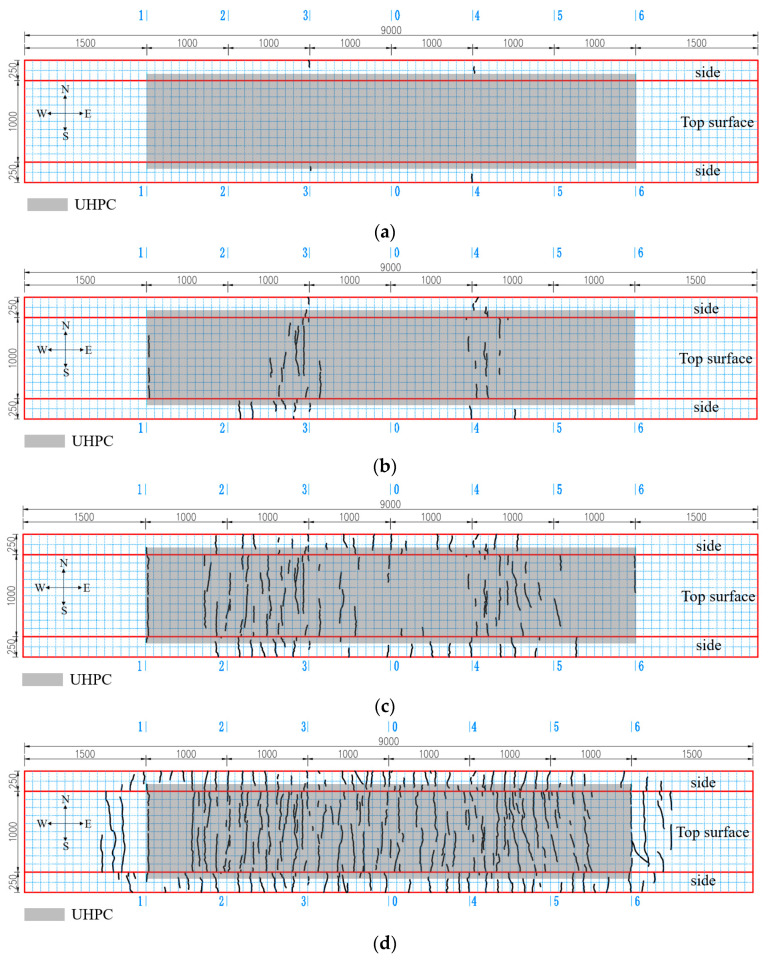
Crack propagation under the load: (**a**) 500 kN; (**b**) 750 kN; (**c**) 1000 kN; and (**d**) 1650 kN.

When the load reached approximately 500 kN, first cracks initiated on the side of the NC layer at the cantilever root sections (section 3 and section 4), with an initial width of 0.03 mm. Immediately after that, the cracks on the side extended into the UHPC layer along the thickness direction. The load corresponding to the initiation of the cracks on the top surface of the UHPC layer was 540 kN, while the load corresponding to a crack width of 0.05 mm was 600 kN. As the load continued to rise, the cracks away from the girder-to-pier joint developed earlier than those within the girder-to-pier joint area. The load corresponding to the initiation of the cracks at the UHPC-NC interface was 750 kN, while the load corresponding to the initiation of the cracks at the central plane of the girder-to-pier joint was 850 kN. The load corresponding to a crack width of 0.2 mm was 1050 kN, which was located on the top surface of the UHPC layer at the cantilever root sections. At the terminal stage of loading, the pullout sound of the steel fibers in the UHPC layer became frequent, and the cracks within the girder-to-pier joint propagated more rapidly. For safety reasons, cracking monitoring was stopped at a load of 1650 kN. As illustrated in Figure 16d, numerous cracks were densely distributed across the specimen, with an average longitudinal spacing of approximately 10 cm.

**Figure 17:**
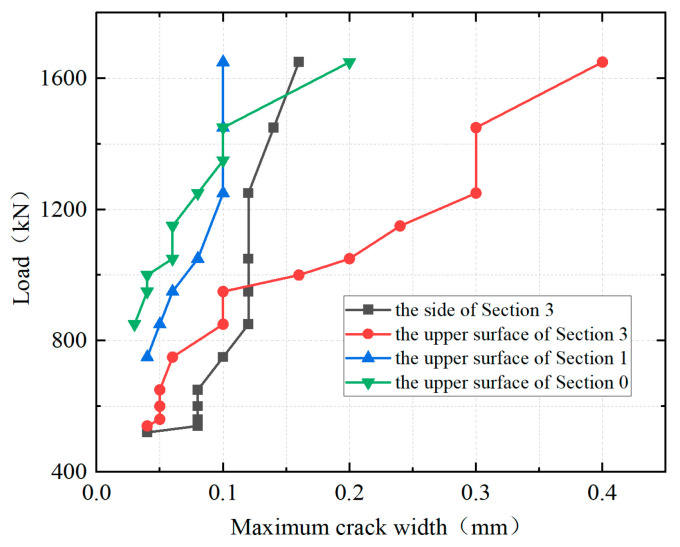
Load-crack width curves.

Figure 17 shows the load-maximum crack width curves of the representative sections. The initial points of the curves indicated that the first cracks initiated on the side of the cantilever root section (section 3), followed by those on the top surface of the UHPC layer of the cantilever root section and then at the UHPC-NC interface (section 1) and the central plane of the girder-to-pier joint (section 0) in sequence. Throughout the whole loading process, the width of the cracks on the side of the cantilever root section and on the top surface of the UHPC-NC interface developed slowly and reached 0.16 mm and 0.1 mm, respectively, at a load of 1650 kN. At the initial stage of loading, the cracks on the top surface of the UHPC layer at Section 3 and Section 0 also developed slowly. However, the cracks at each section began to develop rapidly once their widths reached 0.1 mm, and at a load of 1650 kN, the widths of the cracks were 0.4 mm and 0.2 mm, respectively.

### 4.7. Assessment of Experimental Results

Since this paper employed a full-scale test model of a segment of the hogging moment zone, the specimen could directly reflect the forces experienced by the real bridge at the corresponding positions, enabling the test results to be directly compared with the design value.

#### 4.7.1. Structural Safety Factor

The safety and reliability of the structure depend on the relationship between the load effects and the structural resistance. A structural safety factor is defined as the ratio of the maximum external force that the structure can endure to the actual external force, representing the structure’s level of safety. The design method for bridges is the Limit State Design (LSD), also known as the Load And Resistance Factor Design (LRFD), as expressed in Formula (1), where $S_{G}$ is the dead load effect; $S_{Q}$ is the live load effect; $\gamma_{0}$ is the importance factor of the bridge; $\gamma_{G}$ is the partial factor of the dead load effect; $\gamma_{S}$ is the partial factor of the live load effect; R is the structural resistance, which is the function of the material strength design value $f_{d}$ and the structural geometric parameters $a_{d}$; $f_{y}(f_{u})$ is the material yielding (ultimate) strength; and $\gamma_{R}$ is the partial factor for the material resistance.

(1) $$\gamma_{0}(\gamma_{G}S_{G}+\gamma_{Q}S_{Q})\leq R(f_{d},a_{d})=\frac{R(f_{y}(f_{u}),a_{d})}{\gamma_{R}}$$

As referred to in the literature [28], the proportion of dead load and live load $p_{G}$, $p_{Q}$, and the equivalent partial factor of the load effect $\gamma_{L}$ are introduced to simplify the formula, and the load effects can be expressed as follows: $\gamma_{0}(\gamma_{G}S_{G}+\gamma_{Q}S_{Q})=\gamma_{0}(\gamma_{G}p_{G}+\gamma_{Q}p_{Q})S=\gamma_{0}\gamma_{L}S$, where *S* represents the total load effects. Substituting this into Formula (1) gives Formula (2), and the equivalent safety factor can be defined as Equation (3). Through the above finite element analysis of the overall mechanical performance of the reference engineering, the proportion of the dead load and live load is calculated, and the value of each partial factor is obtained according to the JTGD60-2015 [27]. Finally, the equivalent safety factor of the structure is calculated as 2.3.

(2) $$S\leq \frac{R(f_{y}(f_{u}),a_{d})}{\gamma_{0}\gamma_{R}\gamma_{L}}$$

(3) $$K^{\prime }=\gamma_{0}\gamma_{R}\gamma_{L}$$

**Figure 18:**
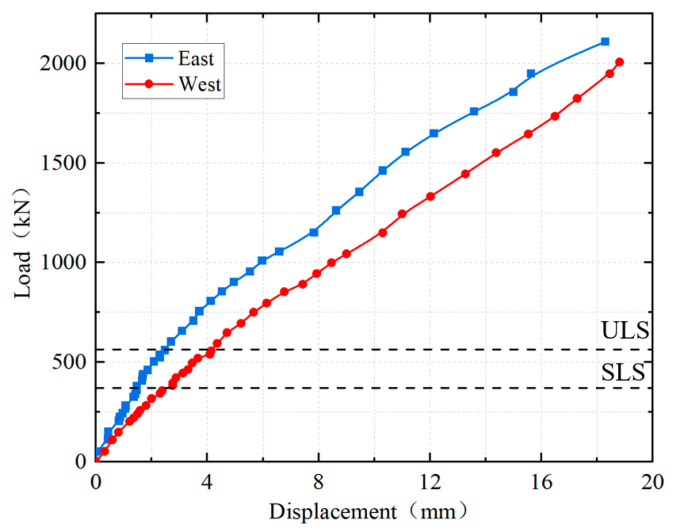
The load corresponding to the serviceability and ultimate limit state of design.

By converting the finite element results using the flexural stiffness equivalent principle, the cantilever root section corresponds to the serviceability limit state of the design at a load of 370.8 kN and to the ultimate limit state at a load of 564.2 kN as shown in Figure 18. The test results indicated that the load corresponding to a crack width of 0.2 mm was 1050 kN. Comparing this test result with the flexural moment of the corresponding section under the serviceability limit state (SLS), as obtained from the finite element analysis, the structural safety factor was determined to be 2.8. When the load reached 2059 kN, the longitudinal reinforcement in the top layer at the cantilever root sections (section 3 and section 4) had just yielded in tension, and the structure reached the edge yield state, at which point the structure was considered damaged. Comparing this test result with the flexural moment of the corresponding section under the ultimate limit state (ULS), the structural safety factor was determined to be 3.6. At this point, only the longitudinal reinforcement in the top layer of section 3 and section 4 had yielded in tension, and the structure still had further capacity before a compression collapse, and the safety factor would be greater than 3.6. Compared with the calculated equivalent safety factor, the flexural capacity of the girder-to-pier joint has been verified.

#### 4.7.2. Nominal Cracking Stress

Cracks in UHPC structures with a width of less than 0.05 mm have only a slight effect on the structural durability [29,30]. Therefore, the load at which the crack width in the UHPC layer reached 0.05 mm was defined as the cracking load *P_cr_* for each section. The nominal cracking stress can be determined using the formula *σ_cr_* = *M_cr_y*/*α_E_I*_0_, where *M_cr_* is the cracking moment of the section; *y* is the distance between the neutral axis and the top surface; *α_E_* = *E_s_*/*E_c_*, the ratio of the elastic modulus of the steel to that of the concrete; and *I*_0_ is the moment of inertia of the converted section of the composite girder. The design stress was the tensile stress of concrete slab under the serviceability limit state (SLS) calculated by the finite element method above. As listed in Table 5, the test values are greater than the design values, indicating that the cracking resistance of this joint meets the requirements.

## 5. Calculation of Elastic Flexural Capacity

It can be observed that the longitudinal reinforcement in the top layer at the cantilever root sections (section 3 and section 4) yielded at the terminal stage of loading, which was identified as the failure mode of the specimen. The elastic flexural capacity of the joint was analyzed using the edge yielding theory based on the above failure mode. Throughout the entire loading process, no obvious slip or separation occurred between the concrete slab and the I-shaped steel girder. The elastic flexural capacity of the girder-to-pier joint can be calculated based on the following fundamental assumptions:

(1) The cross-section of the composite girders conforms to the plane section assumption at all stages;
(2) The tensile strength of the normal concrete is ignored;
(3) The bridging effect of the steel fibers after UHPC cracking is considered, i.e., the contribution of the tensile strength of the UHPC is incorporated;
(4) The contribution of the stud connectors to the moment of inertia of the cross-section is ignored.

**Figure 19:**
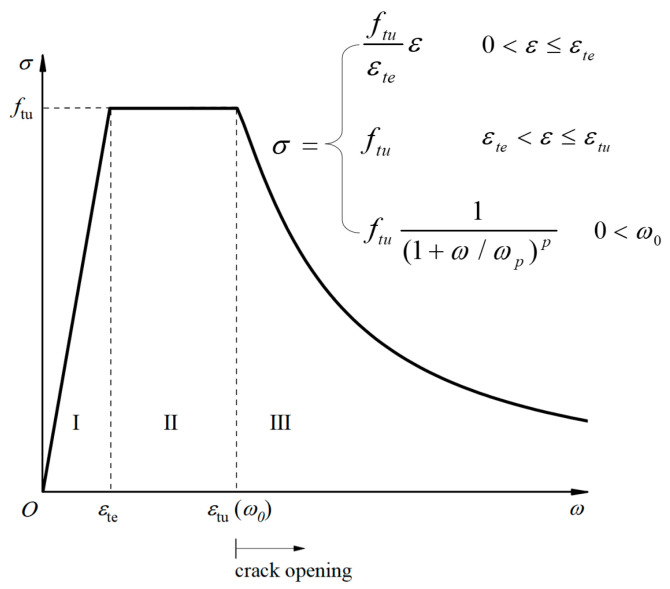
Axial tensile constitutive model of UHPC.

During the elastic flexural capacity calculation stage, the concrete slab had already cracked, and the cross-section consisted of the UHPC layer, the longitudinal reinforcement, and the I-shaped steel girders, as well as the longitudinal reinforcement in the top layer yielded in tension. At this point, the crack width of the UHPC layer at the cantilever root section was substantial and had already entered the stress softening stage. In this paper, the elastic flexural capacity of the joint was calculated based on the axial tensile constitutive model, as referred to in the literature [29]. As shown in Figure 19, the stress–strain curve was applied for stages I and II, and the stress–crack width curve was applied for stage III, where *f_tu_* is the ultimate tensile strength of the UHPC, which was taken to be 7.81 MPa based on the results of the material property test; *ω*_0_ is the corresponding crack width at the peak point; *ω_p_* is the corresponding crack width when the stress is reduced to 2^−p^*f_tu_*; and *p* is the test fitting parameter. As referred to in the literature [31], *ω*_0_ was taken as 0.05 mm, *ω_p_* as 0.52 mm, and *p* as 0.98. Based on the final crack width of the UHPC layer at the cantilever root section, the tensile stress of the UHPC σ_1_ can be determined. A tensile strength coefficient *k* = σ_1_/*f_tu_* was introduced, which was determined to be 0.57.

**Figure 20:**
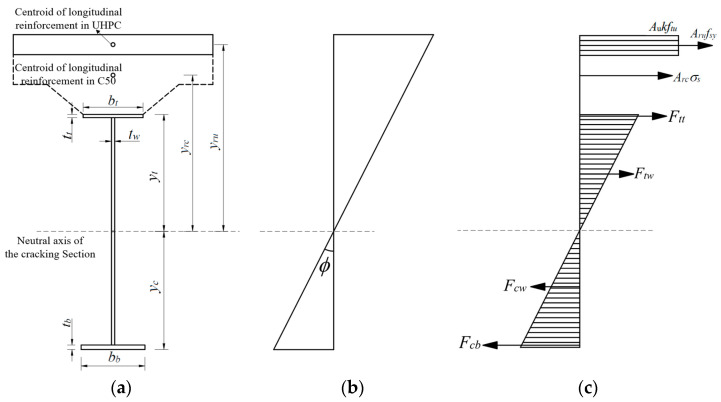
Elastic flexural capacity calculation: (**a**) section parameters; (**b**) strain distribution; and (**c**) stress distribution.

The strain distribution of the cross-section, including curvature and neutral axis position, can be determined using the cross-section axial force equilibrium equation (Equation (4)) according to the graphical calculation shown in Figure 20. Subsequently, the cross-section elastic flexural capacity can be calculated by Equation (5).

*A_u_kf_tu_* + *A_ru_f_sy_* + *A_rc_σ_s_* + *F_tt_* + *F_tw_* = *F_cw_* + *F_cb_* (4)

*M* = *M_tt_* + *M_tw_* + *M_cw_* + *M_cb_* + *A_u_f_tu_y_ur_* + *A_ru_f_sy_y_ru_* + *A_rc_σ_s_y_rc_* (5)

*f_sy_* = *E_s_ϕy_r_* (6)

*F_tt_* = *E_s_ϕt_t_*(*y_t_* − *t_t_*/2)*b_t_* (7)

*M_tt_* = *E_s_ϕb_t_*(*y_t_*^3^ − (*y_t_* − *t_t_*)^3^)/3 (8)

*F_tw_* = *E_s_ϕt_w_*(*y_t_* − *t_t_*)^2^/2 (9)

*M_tw_* = *E_s_ϕt_w_*(*y_t_* − *t_t_*)^3^/3 (10)

*F_cw_* = *E_s_ϕt_w_*(*y_c_* − *t_b_*)^2^/2 (11)

*M_cw_* = *E_s_ϕt_w_*(*y_c_* − *t_b_*)^3^/3 (12)

*F_cb_* = *E_s_ϕt_b_*(*y_c_* − *t_b_*/2)*b_b_* (13)

*M_cb_* = *E_s_ϕb_b_*(*y_c_*^3^ − (*y_c_* − *t_b_*)^3^)/3 (14)

where *A_u_* is the area of the UHPC layer; *f_tu_* is the ultimate tensile strength of UHPC; *k* is the tensile strength coefficient; *A_ru_* is the area of the longitudinal reinforcement in the UHPC layer; *f_sy_* is the yield strength of the longitudinal reinforcement in the UHPC layer; *A_rc_* is the area of the longitudinal reinforcement in the C50 layer; and *σ_s_* is the stress of the longitudinal reinforcement in the C50 layer. *F_tt_* and *F_tw_* are the tensile forces carried by the top flanges and the tensile zone of the web of the steel girder, respectively. *F_cb_* and *F_cw_* are the compressive forces carried by the bottom flanges and the tensile zone of the web of the steel girder, respectively. *M_tt_*, *M_tw_*, *M_cw_*, and *M_cb_* are the flexural moments carried by the top flanges and the tensile zone of the web, the compressive zone of the web, and the bottom flanges of the steel girder, respectively. To enhance clarity for the readers, the first subscript, “*t*”, represents the state of tension; “*c*” represents the state of compression. The second subscript, “*t*”, represents the top flange, “*w*” represents the web, and “*b*” represents the bottom plate. *M* is the elastic flexural capacity of the cross-section; *ϕ* is the section curvature; *E_s_* is the elastic modulus of steel; *y_t_* is the distance between the neutral axis of the cracked section and the top surface of the steel girder; *y_c_* is the distance between the neutral axis of the cracked section and the lower surface of the steel girder; *y_rc_* is the distance between the neutral axis of the cracked section and the centroid of the longitudinal reinforcement in the C50 layer; and *y_ru_* is the distance between the neutral axis of the cracked section and the centroid of the longitudinal reinforcement in the UHPC layer.

The comparison of the elastic flexural capacity of the girder-to-pier joint calculated according to the method above with the experimental results is presented in Table 6. The geometrical parameters of the cross-section and the material properties were based on the actual values. The calculated elastic flexural capacity of this joint was 6661.5 kN·m, with the contribution of the UHPC tensile strength accounting for 5.1%. The experimental and calculated values are in good agreement, with a ratio of 1.02, indicating the feasibility of the proposed calculation method in this paper. Additionally, the elastic flexural capacity was calculated for a scenario in which the thickness of the UHPC layer was 0 mm, setting *f_tu_* to 0 MPa. In this case, the resulting elastic flexural capacity was 6142.0 kN·m, indicating that the application of UHPC can enhance the flexural capacity of this joint, with an increase of 8.5%.

## 6. Conclusions

This paper proposed an innovative girder-to-pier joint for small- and medium-span composite bridges, with the UHPC layer in the hogging moment zone. To verify the crack-resisting and load-resisting capacity of this new joint, a full-scale test was conducted. This paper proposed a calculation method for the elastic flexural capacity of the girder-to-pier joint incorporating the tensile strength of UHPC as well. Based on the above investigations, the following conclusions can be obtained:

(1) The safety factors of this proposed joint at the serviceability and ultimate limit state were 2.8 and 3.6, respectively. Compared with the calculated equivalent safety factor of this bridge, the flexural capacity of the girder-to-pier joint can be verified.
(2) During the loading process, first cracks initiated on the side of the NC layer beneath the UHPC layer. Subsequently, the cracks were observed on the top surface of the UHPC layer, at the interface of UHPC-NC, and at the central plane of the girder-to-pier joint in sequence. At the terminal stage of loading, the cracks within the girder-to-pier joint propagated more rapidly. The final crack distribution was characterized by numerous and dense crack patterns.
(3) This paper also proposes a calculation method for the elastic flexural capacity of the girder-to-pier joint incorporating the tensile strength of UHPC, and the ratio of the test value to the calculated value was 1.02.
(4) The application of UHPC can enhance the flexural capacity of this joint, with an increase of 8.5%.

Through the full-scale static test, the proposed joint demonstrated excellent crack resistance and flexural performance. However, this paper only investigates its short-term static performance under monotonic loading in a normal temperature environment due to the limit of the test conditions. Further research, including the impact of temperature effects, seismic conditions, and the long-term propagation of cracks, is essential for a more comprehensive understanding of the mechanical performance of this joint and its broader application. When it comes to the structural health monitoring and the detection of cracks, an optical energy harvester can be introduced [32].

## Figures and Tables

**Table 1:** Finite element calculation results.

Section	Serviceability Limit State (SLS)		Ultimate Limit State (ULS)	
	Moment (kN·m)	Tensile Stress (MPa)	Moment (kN·m)	Tensile Stress (MPa)
Section I	4157.7	3.8	6454.5	4.6
Section II	5139.8	7.5	7819.3	9.3

**Table 2:** Material properties of steel.

Material	Diameter/Thickness (mm)	*f_y_* (MPa)	*f_u_* (MPa)
reinforcement	20	460	642
	25	415	622
steel plate	16	428	532
	22	433	543

**Table 3:** Material properties of concrete.

Material	*f_tu_* (MPa)	*f_c_* (MPa)	*E_c_* (GPa)
UHPC	7.81	121.3	45.1
C50	/	52.7	34.2

**Table 4:** Material compositions of 1 m^3^ UHPC.

Cement	Fly Ash	Silica Fume	Quartz Sand	Water	Steel Fiber	Water-Reducing Admixture
600	150	100	1275	207	188	26

**Table 5:** Nominal cracking stresses and design stresses in representative sections.

Section	*P_cr_* (kN)	*σ_cr_* (MPa)	Design Stress (MPa)
Section 1	850	4.2	3.8
Section 3	600	7.6	7.5

**Table 6:** Comparison of the test and calculated values of elastic flexural capacity.

y_c_ (mm)	Flexural Capacity (kN·m)	Calculated Load (kN)	Test Load (kN)	Test Load/Calculated Load
766.2	6661.5	2018.6	2058.7	1.02

## Data Availability

The original contributions presented in this study are included in the article. Further inquiries can be directed to the corresponding author.
